# Medical News and Miscellany

**Published:** 1900-02

**Authors:** 


### Medical News and Miscellany.

---

**Dr. C. E. Keller** has removed from Marion to San Antonio.

---

**Dr. J. W. Cole** has removed from Kennedale, to Lakota, Texas.

---

**Dr. A. J. Gray** has removed from Wills Point to Eastland, Texas.

---

**Personal.**—Dr. B. E. Hadra, whose removal from San Antonio to Dallas was mentioned in the JOURNAL a short time ago—took a look at Dallas—and concluded that he would like Waco better, and accordingly changed to Waco.

---

**Twentieth Century Practice.**—The JOURNAL has sixteen volumes of this great work, new, which we will sell for $40—that is, $2.50 per volume; the publisher's price is $5.00 per volume, and sold only on subscription. We don't pay express.

**Dr. E. T Cook,** formerly of Austin and Houston, Texas, but for some years past a prominent practitioner at Hot Springs, Ark., has removed to Staten Island, N. J., where he has opened a hospital for the treatment of morphine habit by a plan of his own.

---

**Dr. T. J. Turpin,** the well known and long-time Quarantine Officer at Laredo—who, when left out by the new administration removed to City of Mexico and entered the Sanitary service of the Republic, has returned to Texas and located at El Paso.

---

**The New Orleans Polyclinic,** thirteenth annual session, opens November 30, 1899; closes May 10, 1900. Every inducement in clinical facilities for those attending. The specialties are fully taught. Further information, New Orleans Polyclinic, New Orleans, La.

---

**The** JOURNAL has excellent authority for saying that "Lithiated Sorghum Comp. (S. & D., see front cover), cures cystitis, reduces prostatic hypertrophy, mitigates and shortens gonorrhea, allays vesical irritability, checks incontinence of urine, increases the flow of urine, makes the urine aseptic, dissolves the uric acid. In brief, it is the *ideal* demulcent diuretic."

---

**Dr. R. Menger,** San Antonio, whose paper illustrated by micro-photographs appears in this issue, and whose spendidly illustrated paper on the same subject as the one herewith, appeared in our September issue, is an amateur,—but the best micro-photographer anywhere. Dr. Menger will photograph microscopic specimens to order, if sent him, charging a reasonable fee.

---

**Blistering** over the fifth dorsal vertebra for hyperemesis gravidarum, as taught by Parvin, has given me most excellent satisfaction. I believe it should be given the prominence of a specific. The mode of applying the remedy is of as much importance as the treatment itself.

DONALD MCKAY.

Houston, Texas, January 24, 1900.

---

**A copy of this issue,** on account of the illustrated article on smallpox, is mailed to every county health officer in Texas. Those who are not already subscribers to the JOURNAL should promptly enroll their names, as the State Health Department will be represented in each issue, and all official papers will appear in the JOURNAL from time to time. Subscription $1 a year, in advance.

---

**Dr. E. Stuver**, formerly of Rawlins, Wyo., removed over a year ago to Fort Collins, Colorado—but notwithstanding the announcement of the change of address, many persons are still writing to him addressing their letters to Rawlins. The doctor will send reprints of his valuable papers, "Mountain Fever," and "Importance of a Knowledge of the Phylogenetic Development of the Child in the Prevention of Children's Diseases," on request.

---

**Dr. J. M. Strayhorn** of Bartlett, Texas, a member of the District Board of Medical Examiners of this district, is attending special courses in surgery and gynecology at the New York Polyclinic and New York Post-Graduate School, and is working with Prof. Wyeth in his private hospital. He will later on attend the polyclinics at Chicago, Baltimore and Philadelphia. He directs us to follow him up with the "Red-Back."

---

**Superintendent Worsham's Report** (Austin), Texas Insane Asylum, for the year ending October 31, 1899, has been received. *The report shows, since the foundation of the Asylum: Admitted, 4098; discharged, 2377; died, 911; remaining, 734.

*[In our January number, third line of the above was omitted by an oversight and the ludicrous error was not discovered till too late to correct it.—DANIEL, Ed.]

---

**The Raven and Dr. Bell:** We have received No. 1 of Vol. V of a neat little publication called *The Raven*—"A literary Magazine of the day for busy doctors." It is published at St. Louis and is owned and edited by Dr. Ralcey Husted Bell, who formerly published the "*Magazine of Medicine*," at Atlanta, Ga. *The Raven* is a successor to that magazine; hence Vol. V. It is most artistically gotten up,—possessing some decidedly novel features in engravings, and is filled with vigorous editorials and beautiful poems. Price of the *Raven* 50 cents a year. Dr. Bell has just issued a volume of poems. We acknowledge the receipt of a copy and will notice it next issue. He will read an original poem at the Medico-Legal Congress at Paris during the exposition this summer.

